# Crystal structure of 5-(1,3-di­thian-2-yl)-2*H*-1,3-benzodioxole

**DOI:** 10.1107/S2056989015002455

**Published:** 2015-02-13

**Authors:** Julio Zukerman-Schpector, Ignez Caracelli, Hélio A. Stefani, Olga Gozhina, Edward R. T. Tiekink

**Affiliations:** a Departmento de Química, Universidade Federal de São Carlos, 13565-905 São Carlos, SP, Brazil; b Departmento de Física, Universidade Federal de São Carlos, 13565-905 São Carlos, SP, Brazil; c Departamento de Farmácia, Faculdade de Ciências Farmacêuticas, Universidade de São Paulo, 05508-900 São Paulo-SP, Brazil; d Department of Chemistry, University of Malaya, 50603 Kuala Lumpur, Malaysia

**Keywords:** crystal structure, 1,3-di­thiane, conformation, 1,3-benzodioxole, C—H⋯π inter­actions

## Abstract

In the title compound, C_11_H_12_O_2_S_2_, two independent but virtually superimposable mol­ecules, *A* and *B*, comprise the asymmetric unit. In each mol­ecule, the 1,3-di­thiane ring has a chair conformation with the 1,4-disposed C atoms being above and below the plane through the remaining four atoms. The substituted benzene ring occupies an equatorial position in each case and forms dihedral angles of 85.62 (9) (mol­ecule *A*) and 85.69 (8)° (mol­ecule *B*) with the least-squares plane through the 1,3-di­thiane ring. The difference between the mol­ecules rests in the conformation of the five-membered 1,3-dioxole ring which is an envelope in mol­ecule *A* (the methyl­ene C atom is the flap) and almost planar in mol­ecule *B* (r.m.s. deviation = 0.046 Å). In the crystal, mol­ecules of *A* self-associate into supra­molecular zigzag chains (generated by glide symmetry along the *c* axis) *via* methyl­ene C—H⋯π inter­actions. Mol­ecules of *B* form similar chains. The chains pack with no specific directional inter­molecular inter­actions between them.

## Related literature   

The title compound has been prepared previously, see: Ballesteros *et al.* (2005 ▸). For the structure of a related compound containing the same mol­ecular skeleton as in the title compound, *i.e.* (19*R*,21*R*,25*S*)-(−)-2-(2-menthyloxy­carb­onyl-3,4-methyl­ene­dioxy­phen­yl)1,3-di­thiane, see: Ratajczak-Sitarz *et al.* (1996 ▸).
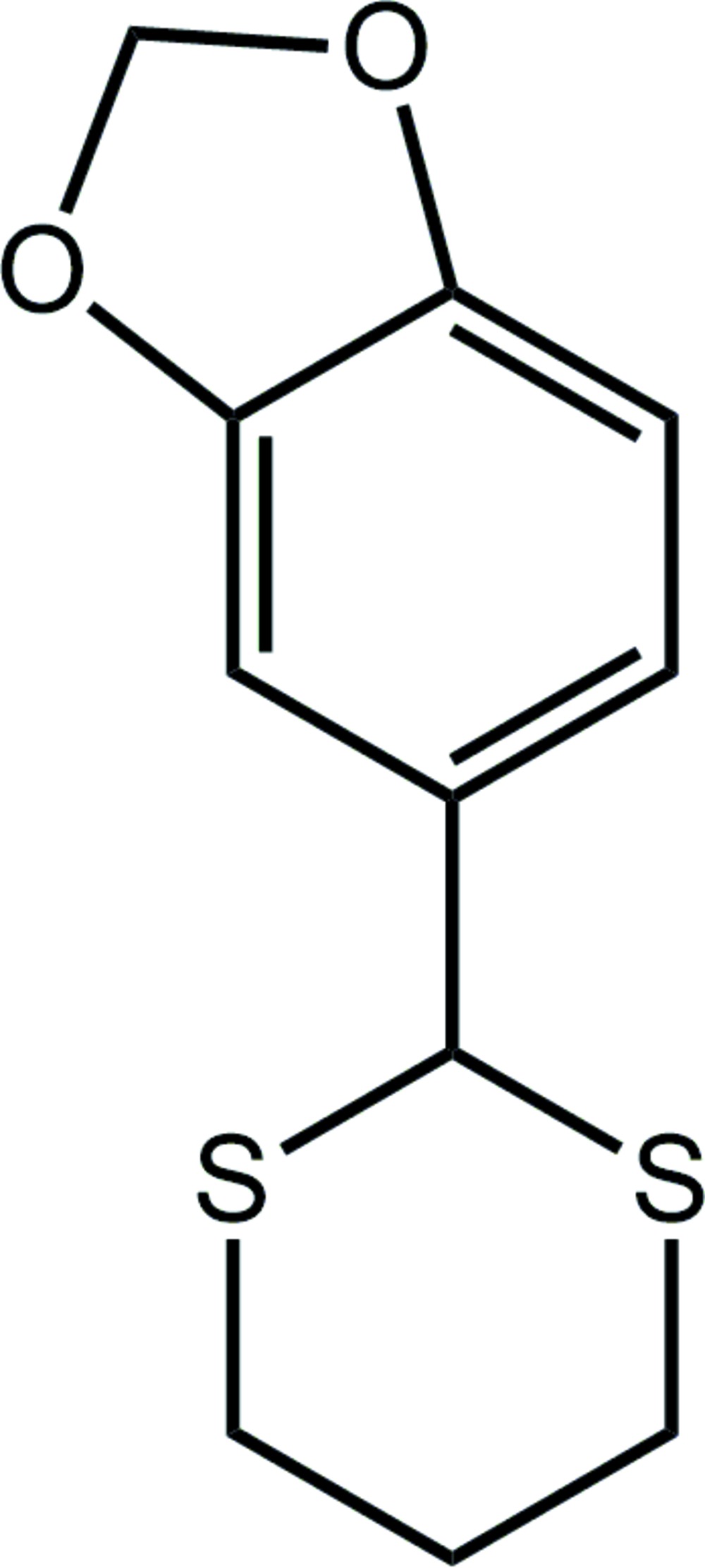


## Experimental   

### Crystal data  

C_11_H_12_O_2_S_2_
*M* *_r_* = 240.33
Monoclinic, 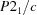

*a* = 11.4765 (3) Å
*b* = 17.5504 (4) Å
*c* = 11.6397 (2) Å
β = 104.275 (1)°
*V* = 2272.05 (9) Å^3^
*Z* = 8
Mo *K*α radiation
μ = 0.45 mm^−1^
*T* = 296 K
0.59 × 0.40 × 0.26 mm

### Data collection  

Bruker APEXII CCD diffractometer
Absorption correction: multi-scan (*SADABS*; Sheldrick, 1996 ▸) *T*_min_ = 0.702, *T*_max_ = 0.745
14839 measured reflections
4164 independent reflections
3759 reflections with *I* > 2σ(*I*)
*R*_int_ = 0.020

### Refinement  

*R*[*F* ^2^ > 2σ(*F* ^2^)] = 0.035
*wR*(*F* ^2^) = 0.092
*S* = 1.03
4164 reflections
271 parameters
H-atom parameters constrained
Δρ_max_ = 0.29 e Å^−3^
Δρ_min_ = −0.44 e Å^−3^

### 

Data collection: *APEX2* (Bruker, 2009 ▸); cell refinement: *SAINT* (Bruker, 2009 ▸); data reduction: *SAINT*; program(s) used to solve structure: *SIR2014* (Burla *et al.*, 2015 ▸); program(s) used to refine structure: *SHELXL2014* (Sheldrick, 2015 ▸); molecular graphics: *ORTEP-3 for Windows* (Farrugia, 2012 ▸), *QMOL* (Gans & Shalloway, 2001 ▸) and *DIAMOND* (Brandenburg, 2006 ▸); software used to prepare material for publication: *MarvinSketch* (ChemAxon, 2010 ▸) and *publCIF* (Westrip, 2010 ▸).

## Supplementary Material

Crystal structure: contains datablock(s) I, New_Global_Publ_Block. DOI: 10.1107/S2056989015002455/hb7361sup1.cif

Structure factors: contains datablock(s) I. DOI: 10.1107/S2056989015002455/hb7361Isup2.hkl

Supporting information file. DOI: 10.1107/S2056989015002455/hb7361Isup3.cml

**Fig. 1.**
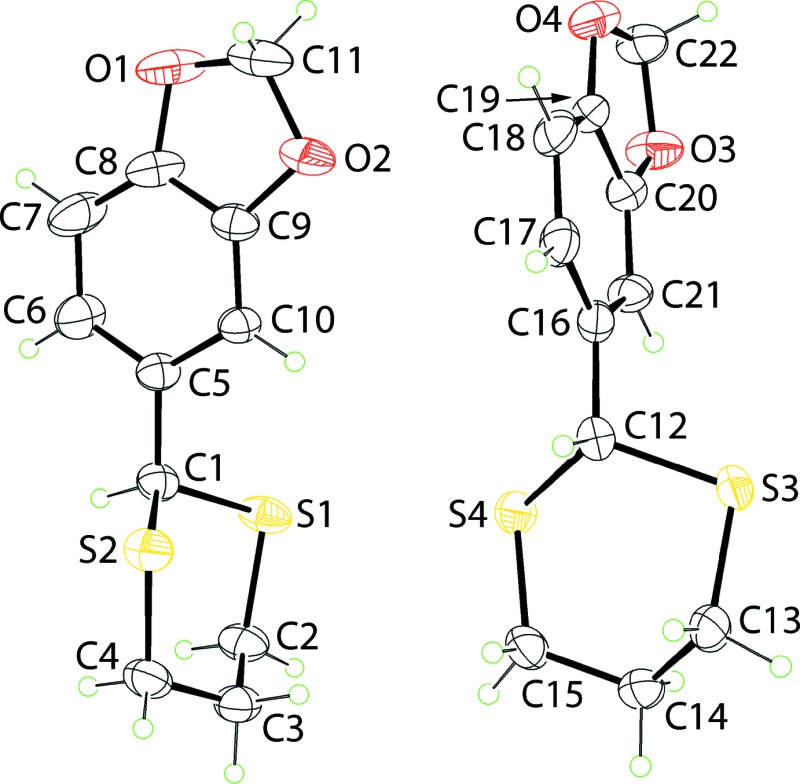
The molecular structures of the two independent molecules in title compound showing the atom-labelling scheme and displacement ellipsoids at the 35% probability level.

. DOI: 10.1107/S2056989015002455/hb7361fig1.tif
The mol­ecular structures of the two independent mol­ecules in title compound showing the atom-labelling scheme and displacement ellipsoids at the 35% probability level.

**Fig. 2.**
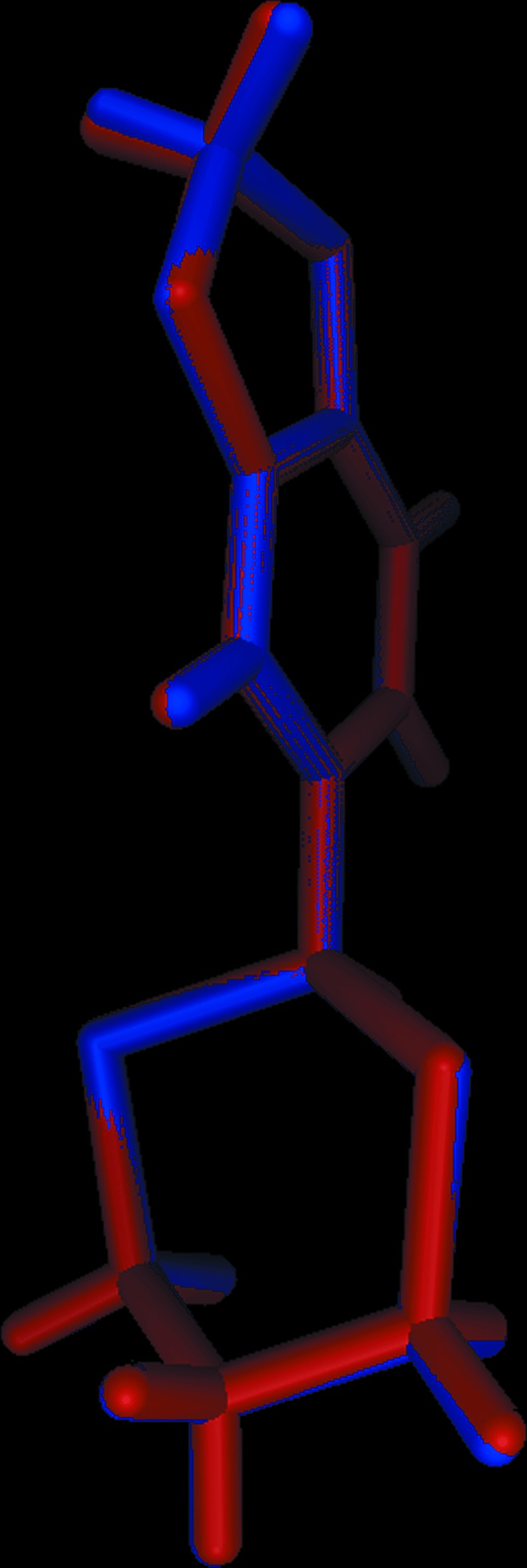
Superimposition of the two independent molecules. Molecule A is shown in red and B in blue. The molecules have been superimposed such that the benzene rings are overlapped.

A B . DOI: 10.1107/S2056989015002455/hb7361fig2.tif
Superimposition of the two independent mol­ecules. Mol­ecule *A* is shown in red and *B* in blue. The mol­ecules have been superimposed such that the benzene rings are overlapped.

**Fig. 3.**
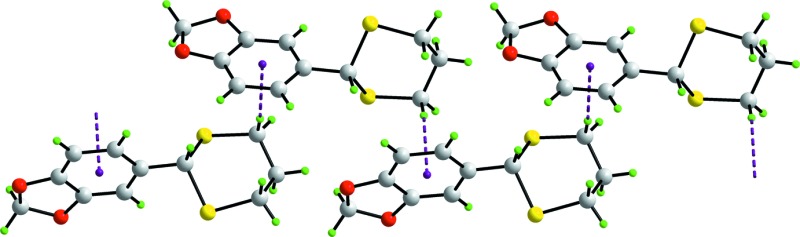
A view of the zigzag supramolecular chain comprising molecules of A along the c axis (glide symmetry) mediated by C—H···π interactions are shown as purple dashed lines.

**Fig. 4.**
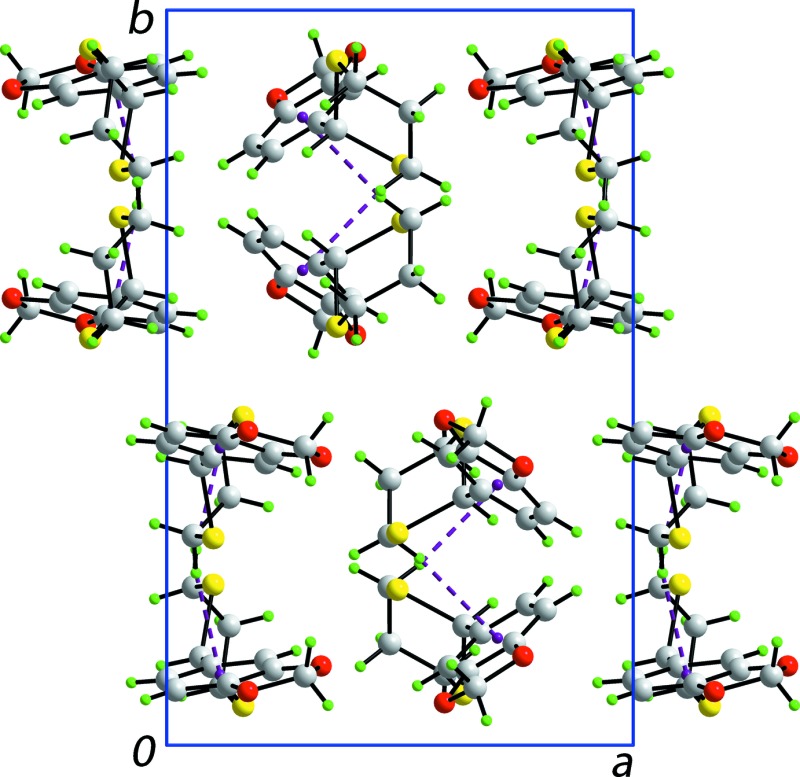
A view in projection down the c axis of the unit-cell contents. The C—H···π interactions are shown as purple dashed lines.

A c . DOI: 10.1107/S2056989015002455/hb7361fig3.tif
A view of the zigzag supra­molecular chain comprising mol­ecules of *A* along the *c* axis (glide symmetry) mediated by C—H⋯π inter­actions are shown as purple dashed lines.

c . DOI: 10.1107/S2056989015002455/hb7361fig4.tif
A view in projection down the *c* axis of the unit-cell contents. The C—H⋯π inter­actions are shown as purple dashed lines.

CCDC reference: 1047484

Additional supporting information:  crystallographic information; 3D view; checkCIF report

## Figures and Tables

**Table 1:** Hydrogen-bond geometry (, ) *Cg*1 and *Cg*2 are the centroids of the C5C11 and C16C21 rings, respectively.

*D*H*A*	*D*H	H*A*	*D* *A*	*D*H*A*
C4H4b*Cg*1^i^	0.97	2.77	3.731(2)	170
C13H13a*Cg*2^ii^	0.97	2.54	3.4841(19)	165

Symmetry codes: (i) 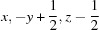; (ii) 
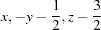
.

## supplementary crystallographic information

### S1. Experimental

A solution of the corresponding 2*H*-1,3-benzodioxole-5-carbaldehyde (0.037 mol, 1 equiv.) in chloroform (20 ml) was combined with an equimolar amount of propane-1,3-dithiol (3.7 ml, 0.037 mol) at room temperature. The solution was stirred for 1 h at this temperature, then cooled to -20 °C after which BF_3_ etherate (0.46 ml, 0.0037 mol, 0.1 equiv.) was added drop-wise. The reaction solution was allowed to warm to room temperature and stirred overnight. After this time, the solution was washed three times each with water, 10% aqueous KOH, then water followed by drying over MgSO_4_. Evaporation of the solvent furnishes a pure product as colourless crystals in 97% yield. To obtain crystals suitable for X-ray analysis, the product was crystallized from CH_3_OH. The spectroscopic data matched those reported in the literature (Ballesteros *et al.*, 2005).

### S2. Refinement

Carbon-bound H-atoms were placed in calculated positions (C—H = 0.93–0.98 Å) and were included in the refinement in the riding model approximation, with *U*_iso_(H) = 1.2 *U*_eq_(C).

### Crystal data

**Table:**

C_11_H_12_O_2_S_2_	*F*(000) = 1008
*M**_r_* = 240.33	*D*_x_ = 1.405 Mg m^−^^3^
Monoclinic, *P*2_1_/*c*	Mo *K*α radiation, λ = 0.71073 Å
*a* = 11.4765 (3) Å	Cell parameters from 9068 reflections
*b* = 17.5504 (4) Å	θ = 2.9–25.4°
*c* = 11.6397 (2) Å	µ = 0.45 mm^−^^1^
β = 104.275 (1)°	*T* = 296 K
*V* = 2272.05 (9) Å^3^	Prism, colourless
*Z* = 8	0.59 × 0.40 × 0.26 mm

### Data collection

**Table:**

Bruker APEXII CCD diffractometer	3759 reflections with *I* > 2σ(*I*)
φ and ω scans	*R*_int_ = 0.020
Absorption correction: multi-scan (*SADABS*; Sheldrick, 1996)	θ_max_ = 25.4°, θ_min_ = 1.8°
*T*_min_ = 0.702, *T*_max_ = 0.745	*h* = −13→13
14839 measured reflections	*k* = −21→19
4164 independent reflections	*l* = −14→8

### Refinement

**Table:**

Refinement on *F*^2^	0 restraints
Least-squares matrix: full	Hydrogen site location: inferred from neighbouring sites
*R*[*F*^2^ > 2σ(*F*^2^)] = 0.035	H-atom parameters constrained
*wR*(*F*^2^) = 0.092	*w* = 1/[σ^2^(*F*_o_^2^) + (0.0459*P*)^2^ + 0.9363*P*] where *P* = (*F*_o_^2^ + 2*F*_c_^2^)/3
*S* = 1.03	(Δ/σ)_max_ = 0.001
4164 reflections	Δρ_max_ = 0.29 e Å^−^^3^
271 parameters	Δρ_min_ = −0.44 e Å^−^^3^

### Special details

**Table:**

Geometry. All e.s.d.'s (except the e.s.d. in the dihedral angle between two l.s. planes) are estimated using the full covariance matrix. The cell e.s.d.'s are taken into account individually in the estimation of e.s.d.'s in distances, angles and torsion angles; correlations between e.s.d.'s in cell parameters are only used when they are defined by crystal symmetry. An approximate (isotropic) treatment of cell e.s.d.'s is used for estimating e.s.d.'s involving l.s. planes.

### Fractional atomic coordinates and isotropic or equivalent isotropic displacement parameters (Å^2^)

**Table:**

	*x*	*y*	*z*	*U*_iso_*/*U*_eq_	
S1	0.36505 (5)	0.56934 (3)	0.82081 (4)	0.05661 (16)	
S2	0.50542 (5)	0.71160 (3)	0.79700 (4)	0.05188 (15)	
O1	0.23644 (16)	0.62085 (13)	0.26070 (13)	0.0865 (6)	
O2	0.40467 (15)	0.55784 (9)	0.36797 (12)	0.0660 (4)	
C1	0.36233 (15)	0.66240 (10)	0.75248 (15)	0.0432 (4)	
H1	0.3013	0.6935	0.7762	0.052*	
C2	0.4034 (2)	0.59674 (13)	0.97523 (16)	0.0553 (5)	
H2A	0.3401	0.6291	0.9899	0.066*	
H2B	0.4066	0.5513	1.0234	0.066*	
C3	0.52212 (18)	0.63848 (12)	1.01417 (16)	0.0524 (5)	
H3A	0.5408	0.6455	1.0994	0.063*	
H3B	0.5849	0.6071	0.9962	0.063*	
C4	0.5228 (2)	0.71522 (12)	0.95595 (17)	0.0567 (5)	
H4A	0.5980	0.7406	0.9919	0.068*	
H4B	0.4583	0.7459	0.9720	0.068*	
C5	0.32654 (15)	0.65344 (11)	0.62010 (15)	0.0428 (4)	
C6	0.22667 (18)	0.69163 (15)	0.55466 (19)	0.0640 (6)	
H6	0.1835	0.7230	0.5937	0.077*	
C7	0.1889 (2)	0.68440 (18)	0.4319 (2)	0.0779 (7)	
H7	0.1215	0.7100	0.3882	0.094*	
C8	0.25501 (19)	0.63842 (14)	0.37937 (16)	0.0602 (6)	
C9	0.35527 (17)	0.60054 (11)	0.44322 (15)	0.0464 (4)	
C10	0.39414 (16)	0.60659 (10)	0.56338 (15)	0.0426 (4)	
H10	0.4621	0.5809	0.6056	0.051*	
C11	0.3389 (3)	0.57881 (16)	0.25229 (18)	0.0757 (7)	
H11A	0.3891	0.6094	0.2143	0.091*	
H11B	0.3145	0.5335	0.2047	0.091*	
S3	0.09983 (5)	0.28175 (2)	0.03890 (4)	0.04927 (14)	
S4	0.16401 (4)	0.44631 (2)	0.00810 (4)	0.04651 (14)	
O3	0.33099 (13)	0.39219 (10)	0.48171 (12)	0.0623 (4)	
O4	0.17354 (14)	0.42693 (9)	0.56131 (12)	0.0626 (4)	
C12	0.07298 (15)	0.38075 (9)	0.06991 (15)	0.0393 (4)	
H12	−0.0118	0.3922	0.0342	0.047*	
C13	0.05738 (19)	0.28499 (10)	−0.12132 (16)	0.0496 (4)	
H13A	0.0628	0.2340	−0.1517	0.060*	
H13B	−0.0258	0.3011	−0.1470	0.060*	
C14	0.13363 (18)	0.33772 (11)	−0.17442 (16)	0.0484 (4)	
H14A	0.1138	0.3306	−0.2597	0.058*	
H14B	0.2176	0.3245	−0.1438	0.058*	
C15	0.11586 (19)	0.42070 (10)	−0.14730 (16)	0.0504 (5)	
H15A	0.0312	0.4330	−0.1756	0.060*	
H15B	0.1596	0.4518	−0.1914	0.060*	
C16	0.09597 (16)	0.39253 (9)	0.20158 (15)	0.0401 (4)	
C17	0.00141 (17)	0.41118 (10)	0.25062 (18)	0.0460 (4)	
H17	−0.0757	0.4149	0.2014	0.055*	
C18	0.01841 (18)	0.42465 (11)	0.37213 (18)	0.0521 (5)	
H18	−0.0452	0.4381	0.4044	0.063*	
C19	0.13232 (18)	0.41716 (10)	0.44076 (16)	0.0451 (4)	
C20	0.22716 (16)	0.39691 (10)	0.39325 (16)	0.0435 (4)	
C21	0.21260 (16)	0.38462 (11)	0.27471 (15)	0.0441 (4)	
H21	0.2772	0.3716	0.2437	0.053*	
C22	0.2965 (2)	0.40546 (14)	0.58912 (18)	0.0616 (5)	
H22A	0.3083	0.3596	0.6371	0.074*	
H22B	0.3455	0.4457	0.6338	0.074*	

### Atomic displacement parameters (Å^2^)

**Table:**

	*U*^11^	*U*^22^	*U*^33^	*U*^12^	*U*^13^	*U*^23^
S1	0.0779 (4)	0.0577 (3)	0.0369 (3)	−0.0258 (3)	0.0194 (2)	−0.0058 (2)
S2	0.0583 (3)	0.0509 (3)	0.0473 (3)	−0.0141 (2)	0.0146 (2)	−0.0003 (2)
O1	0.0774 (11)	0.1433 (18)	0.0318 (7)	−0.0069 (11)	0.0003 (7)	−0.0008 (9)
O2	0.0837 (11)	0.0812 (11)	0.0364 (7)	0.0008 (8)	0.0209 (7)	−0.0108 (7)
C1	0.0412 (9)	0.0521 (10)	0.0391 (9)	0.0008 (8)	0.0155 (7)	−0.0036 (8)
C2	0.0696 (13)	0.0651 (13)	0.0352 (9)	−0.0103 (10)	0.0208 (9)	−0.0019 (9)
C3	0.0580 (12)	0.0621 (12)	0.0359 (9)	−0.0002 (9)	0.0092 (8)	−0.0045 (8)
C4	0.0662 (13)	0.0545 (12)	0.0459 (11)	−0.0100 (10)	0.0068 (9)	−0.0122 (9)
C5	0.0380 (9)	0.0554 (11)	0.0370 (9)	−0.0024 (8)	0.0130 (7)	0.0002 (8)
C6	0.0459 (11)	0.0932 (17)	0.0539 (12)	0.0180 (11)	0.0142 (9)	0.0020 (11)
C7	0.0512 (12)	0.124 (2)	0.0516 (12)	0.0217 (14)	−0.0001 (10)	0.0118 (13)
C8	0.0507 (11)	0.0919 (16)	0.0343 (10)	−0.0106 (11)	0.0032 (8)	0.0042 (10)
C9	0.0516 (10)	0.0543 (11)	0.0358 (9)	−0.0091 (8)	0.0155 (8)	−0.0023 (8)
C10	0.0439 (9)	0.0509 (10)	0.0335 (8)	0.0003 (8)	0.0104 (7)	0.0015 (7)
C11	0.0981 (19)	0.0934 (18)	0.0356 (11)	−0.0223 (15)	0.0166 (11)	−0.0127 (11)
S3	0.0712 (3)	0.0321 (2)	0.0429 (3)	−0.0009 (2)	0.0108 (2)	0.00570 (17)
S4	0.0588 (3)	0.0368 (2)	0.0442 (3)	−0.01144 (19)	0.0132 (2)	−0.00229 (18)
O3	0.0544 (8)	0.0930 (11)	0.0390 (7)	0.0080 (7)	0.0104 (6)	−0.0048 (7)
O4	0.0798 (10)	0.0687 (9)	0.0452 (8)	0.0103 (8)	0.0265 (7)	−0.0080 (7)
C12	0.0401 (9)	0.0347 (8)	0.0426 (9)	0.0011 (7)	0.0091 (7)	0.0036 (7)
C13	0.0643 (12)	0.0356 (9)	0.0443 (10)	−0.0069 (8)	0.0046 (9)	−0.0028 (7)
C14	0.0598 (11)	0.0477 (10)	0.0362 (9)	−0.0050 (8)	0.0092 (8)	−0.0039 (8)
C15	0.0677 (12)	0.0404 (10)	0.0420 (10)	−0.0090 (9)	0.0111 (9)	0.0061 (8)
C16	0.0447 (9)	0.0330 (8)	0.0448 (9)	0.0036 (7)	0.0155 (8)	0.0024 (7)
C17	0.0434 (9)	0.0388 (9)	0.0578 (11)	0.0092 (7)	0.0161 (8)	0.0032 (8)
C18	0.0550 (11)	0.0453 (10)	0.0654 (13)	0.0117 (8)	0.0325 (10)	−0.0021 (9)
C19	0.0623 (12)	0.0348 (9)	0.0440 (10)	0.0037 (8)	0.0240 (9)	−0.0034 (7)
C20	0.0460 (10)	0.0436 (10)	0.0424 (9)	0.0032 (8)	0.0136 (8)	0.0000 (7)
C21	0.0411 (9)	0.0535 (11)	0.0412 (9)	0.0062 (8)	0.0169 (8)	−0.0004 (8)
C22	0.0757 (15)	0.0689 (14)	0.0411 (10)	−0.0015 (11)	0.0162 (10)	−0.0090 (10)

### Geometric parameters (Å, º)

**Table:**

S1—C2	1.8071 (18)	S3—C13	1.8084 (18)
S1—C1	1.8136 (19)	S3—C12	1.8163 (17)
S2—C4	1.812 (2)	S4—C15	1.8128 (19)
S2—C1	1.8142 (18)	S4—C12	1.8176 (17)
O1—C8	1.380 (2)	O3—C20	1.372 (2)
O1—C11	1.412 (3)	O3—C22	1.420 (2)
O2—C9	1.377 (2)	O4—C19	1.377 (2)
O2—C11	1.420 (3)	O4—C22	1.419 (3)
C1—C5	1.502 (2)	C12—C16	1.504 (2)
C1—H1	0.9800	C12—H12	0.9800
C2—C3	1.515 (3)	C13—C14	1.507 (3)
C2—H2A	0.9700	C13—H13A	0.9700
C2—H2B	0.9700	C13—H13B	0.9700
C3—C4	1.509 (3)	C14—C15	1.515 (3)
C3—H3A	0.9700	C14—H14A	0.9700
C3—H3B	0.9700	C14—H14B	0.9700
C4—H4A	0.9700	C15—H15A	0.9700
C4—H4B	0.9700	C15—H15B	0.9700
C5—C6	1.383 (3)	C16—C17	1.384 (2)
C5—C10	1.402 (2)	C16—C21	1.405 (2)
C6—C7	1.393 (3)	C17—C18	1.399 (3)
C6—H6	0.9300	C17—H17	0.9300
C7—C8	1.353 (3)	C18—C19	1.360 (3)
C7—H7	0.9300	C18—H18	0.9300
C8—C9	1.377 (3)	C19—C20	1.383 (2)
C9—C10	1.363 (2)	C20—C21	1.365 (2)
C10—H10	0.9300	C21—H21	0.9300
C11—H11A	0.9700	C22—H22A	0.9700
C11—H11B	0.9700	C22—H22B	0.9700
			
C2—S1—C1	99.80 (9)	C13—S3—C12	99.16 (8)
C4—S2—C1	99.93 (9)	C15—S4—C12	100.05 (8)
C8—O1—C11	105.10 (17)	C20—O3—C22	105.77 (15)
C9—O2—C11	104.86 (18)	C19—O4—C22	105.78 (14)
C5—C1—S1	109.05 (13)	C16—C12—S3	109.78 (11)
C5—C1—S2	110.02 (12)	C16—C12—S4	110.05 (12)
S1—C1—S2	112.76 (10)	S3—C12—S4	112.62 (9)
C5—C1—H1	108.3	C16—C12—H12	108.1
S1—C1—H1	108.3	S3—C12—H12	108.1
S2—C1—H1	108.3	S4—C12—H12	108.1
C3—C2—S1	114.00 (13)	C14—C13—S3	113.97 (13)
C3—C2—H2A	108.8	C14—C13—H13A	108.8
S1—C2—H2A	108.8	S3—C13—H13A	108.8
C3—C2—H2B	108.8	C14—C13—H13B	108.8
S1—C2—H2B	108.8	S3—C13—H13B	108.8
H2A—C2—H2B	107.6	H13A—C13—H13B	107.7
C4—C3—C2	113.67 (17)	C13—C14—C15	112.54 (16)
C4—C3—H3A	108.8	C13—C14—H14A	109.1
C2—C3—H3A	108.8	C15—C14—H14A	109.1
C4—C3—H3B	108.8	C13—C14—H14B	109.1
C2—C3—H3B	108.8	C15—C14—H14B	109.1
H3A—C3—H3B	107.7	H14A—C14—H14B	107.8
C3—C4—S2	114.57 (13)	C14—C15—S4	115.01 (13)
C3—C4—H4A	108.6	C14—C15—H15A	108.5
S2—C4—H4A	108.6	S4—C15—H15A	108.5
C3—C4—H4B	108.6	C14—C15—H15B	108.5
S2—C4—H4B	108.6	S4—C15—H15B	108.5
H4A—C4—H4B	107.6	H15A—C15—H15B	107.5
C6—C5—C10	120.29 (17)	C17—C16—C21	120.01 (16)
C6—C5—C1	119.61 (17)	C17—C16—C12	119.63 (16)
C10—C5—C1	120.09 (16)	C21—C16—C12	120.36 (15)
C5—C6—C7	121.8 (2)	C16—C17—C18	121.92 (18)
C5—C6—H6	119.1	C16—C17—H17	119.0
C7—C6—H6	119.1	C18—C17—H17	119.0
C8—C7—C6	116.8 (2)	C19—C18—C17	116.85 (16)
C8—C7—H7	121.6	C19—C18—H18	121.6
C6—C7—H7	121.6	C17—C18—H18	121.6
C7—C8—C9	122.08 (18)	C18—C19—O4	128.74 (17)
C7—C8—O1	128.4 (2)	C18—C19—C20	121.81 (17)
C9—C8—O1	109.6 (2)	O4—C19—C20	109.45 (17)
C10—C9—O2	127.83 (18)	C21—C20—O3	128.03 (16)
C10—C9—C8	122.31 (18)	C21—C20—C19	122.16 (17)
O2—C9—C8	109.86 (16)	O3—C20—C19	109.81 (16)
C9—C10—C5	116.72 (17)	C20—C21—C16	117.23 (16)
C9—C10—H10	121.6	C20—C21—H21	121.4
C5—C10—H10	121.6	C16—C21—H21	121.4
O1—C11—O2	109.06 (18)	O4—C22—O3	108.66 (16)
O1—C11—H11A	109.9	O4—C22—H22A	110.0
O2—C11—H11A	109.9	O3—C22—H22A	110.0
O1—C11—H11B	109.9	O4—C22—H22B	110.0
O2—C11—H11B	109.9	O3—C22—H22B	110.0
H11A—C11—H11B	108.3	H22A—C22—H22B	108.3
			
C2—S1—C1—C5	−177.74 (12)	C13—S3—C12—C16	176.91 (13)
C2—S1—C1—S2	59.74 (11)	C13—S3—C12—S4	−60.10 (11)
C4—S2—C1—C5	179.08 (13)	C15—S4—C12—C16	−178.87 (12)
C4—S2—C1—S1	−58.95 (11)	C15—S4—C12—S3	58.30 (11)
C1—S1—C2—C3	−59.52 (17)	C12—S3—C13—C14	61.79 (16)
S1—C2—C3—C4	65.6 (2)	S3—C13—C14—C15	−67.4 (2)
C2—C3—C4—S2	−64.9 (2)	C13—C14—C15—S4	65.0 (2)
C1—S2—C4—C3	58.13 (18)	C12—S4—C15—C14	−57.47 (16)
S1—C1—C5—C6	122.30 (18)	S3—C12—C16—C17	−113.76 (16)
S2—C1—C5—C6	−113.55 (18)	S4—C12—C16—C17	121.74 (15)
S1—C1—C5—C10	−57.71 (19)	S3—C12—C16—C21	66.16 (19)
S2—C1—C5—C10	66.4 (2)	S4—C12—C16—C21	−58.33 (19)
C10—C5—C6—C7	0.9 (3)	C21—C16—C17—C18	1.7 (3)
C1—C5—C6—C7	−179.2 (2)	C12—C16—C17—C18	−178.35 (16)
C5—C6—C7—C8	−0.3 (4)	C16—C17—C18—C19	−1.2 (3)
C6—C7—C8—C9	−0.3 (4)	C17—C18—C19—O4	−179.94 (18)
C6—C7—C8—O1	179.1 (2)	C17—C18—C19—C20	−0.2 (3)
C11—O1—C8—C7	172.7 (3)	C22—O4—C19—C18	174.8 (2)
C11—O1—C8—C9	−7.8 (3)	C22—O4—C19—C20	−5.0 (2)
C11—O2—C9—C10	−173.3 (2)	C22—O3—C20—C21	−176.4 (2)
C11—O2—C9—C8	7.3 (2)	C22—O3—C20—C19	3.8 (2)
C7—C8—C9—C10	0.3 (3)	C18—C19—C20—C21	1.2 (3)
O1—C8—C9—C10	−179.17 (18)	O4—C19—C20—C21	−179.04 (17)
C7—C8—C9—O2	179.8 (2)	C18—C19—C20—O3	−178.97 (17)
O1—C8—C9—O2	0.3 (2)	O4—C19—C20—O3	0.8 (2)
O2—C9—C10—C5	−179.15 (18)	O3—C20—C21—C16	179.54 (18)
C8—C9—C10—C5	0.2 (3)	C19—C20—C21—C16	−0.7 (3)
C6—C5—C10—C9	−0.8 (3)	C17—C16—C21—C20	−0.7 (3)
C1—C5—C10—C9	179.21 (16)	C12—C16—C21—C20	179.33 (16)
C8—O1—C11—O2	12.4 (3)	C19—O4—C22—O3	7.3 (2)
C9—O2—C11—O1	−12.2 (2)	C20—O3—C22—O4	−6.8 (2)

### Hydrogen-bond geometry (Å, º)

Cg1 and Cg2 are the centroids of the C5–C11 and C16–C21 rings, respectively.

**Table:**

*D*—H···*A*	*D*—H	H···*A*	*D*···*A*	*D*—H···*A*
C4—H4b···*Cg*1^i^	0.97	2.77	3.731 (2)	170
C13—H13a···*Cg*2^ii^	0.97	2.54	3.4841 (19)	165

Symmetry codes: (i) *x*, −*y*+1/2, *z*−1/2; (ii) *x*, −*y*−1/2, *z*−3/2.
