# Supplementary figures and images for: Crystal structure of 5-(1,3-di­thian-2-yl)-2H-1,3-benzodioxole
Source: Acta Crystallogr E Crystallogr Commun. 2015 Feb 13;71(Pt 3):o167–8. doi: 10.1107/S2056989015002455 (PMC4350690; doi:10.1107/S2056989015002455)

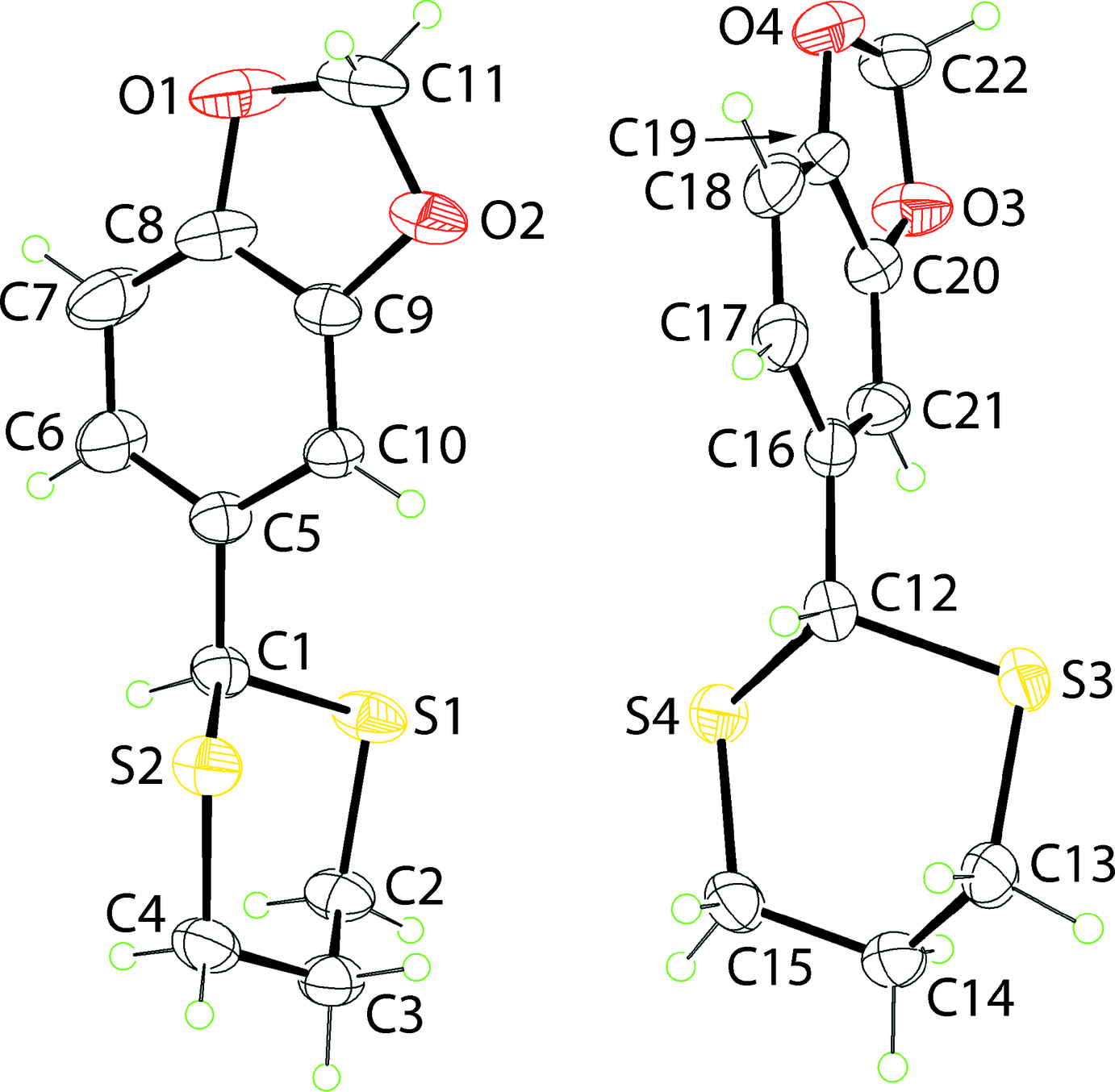

Supplement: Supplementary file 4 [file e-71-0o167-fig1.tif]

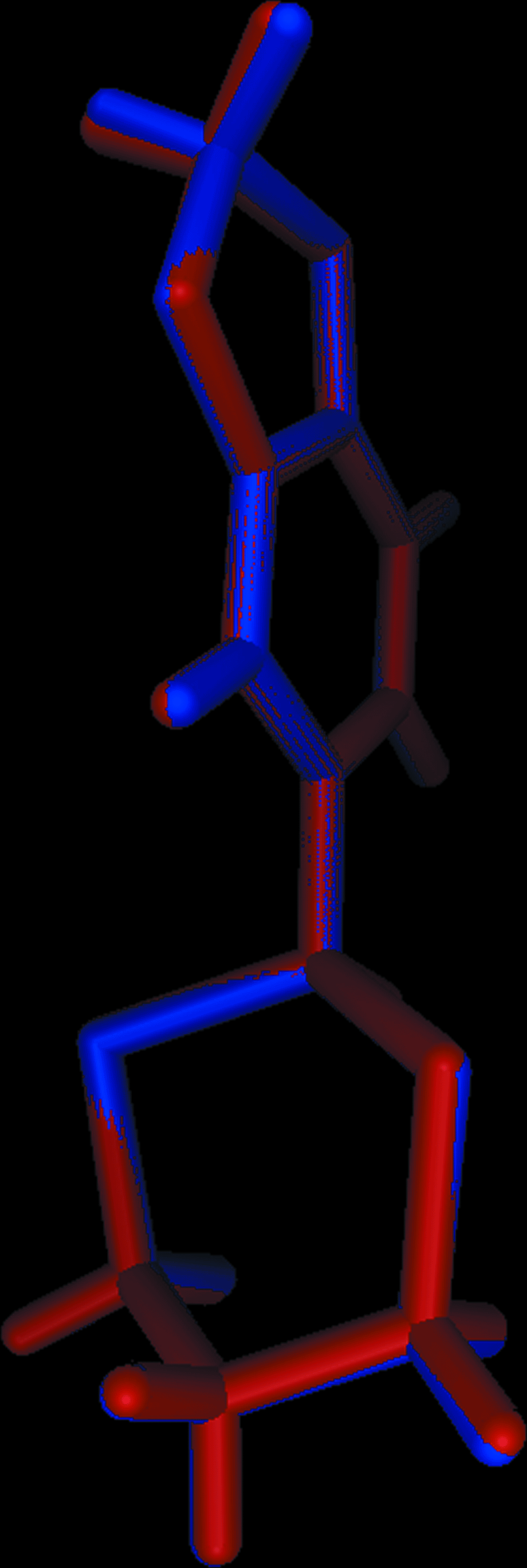

Supplement: Supplementary file 5 [file e-71-0o167-fig2.tif]

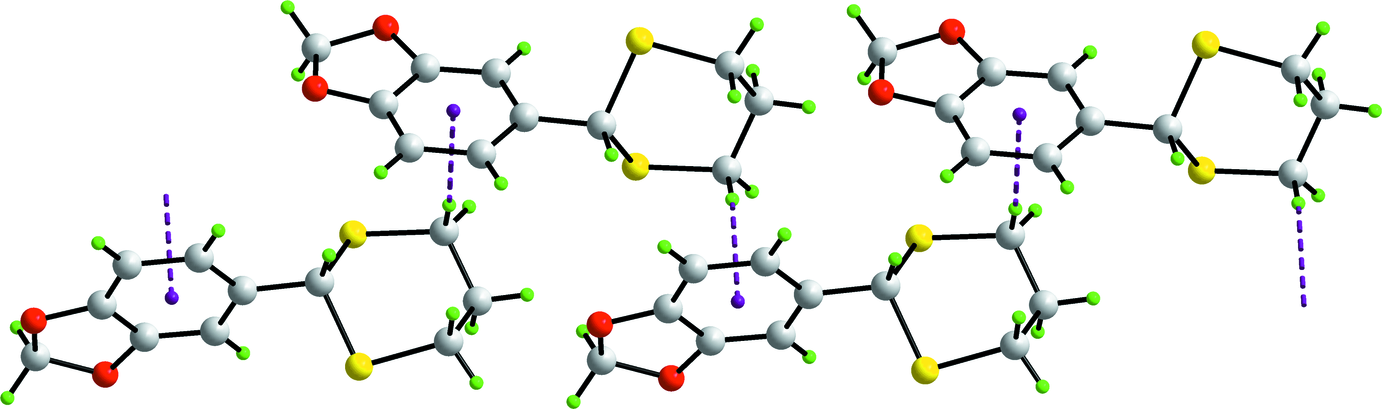

Supplement: Supplementary file 6 [file e-71-0o167-fig3.tif]

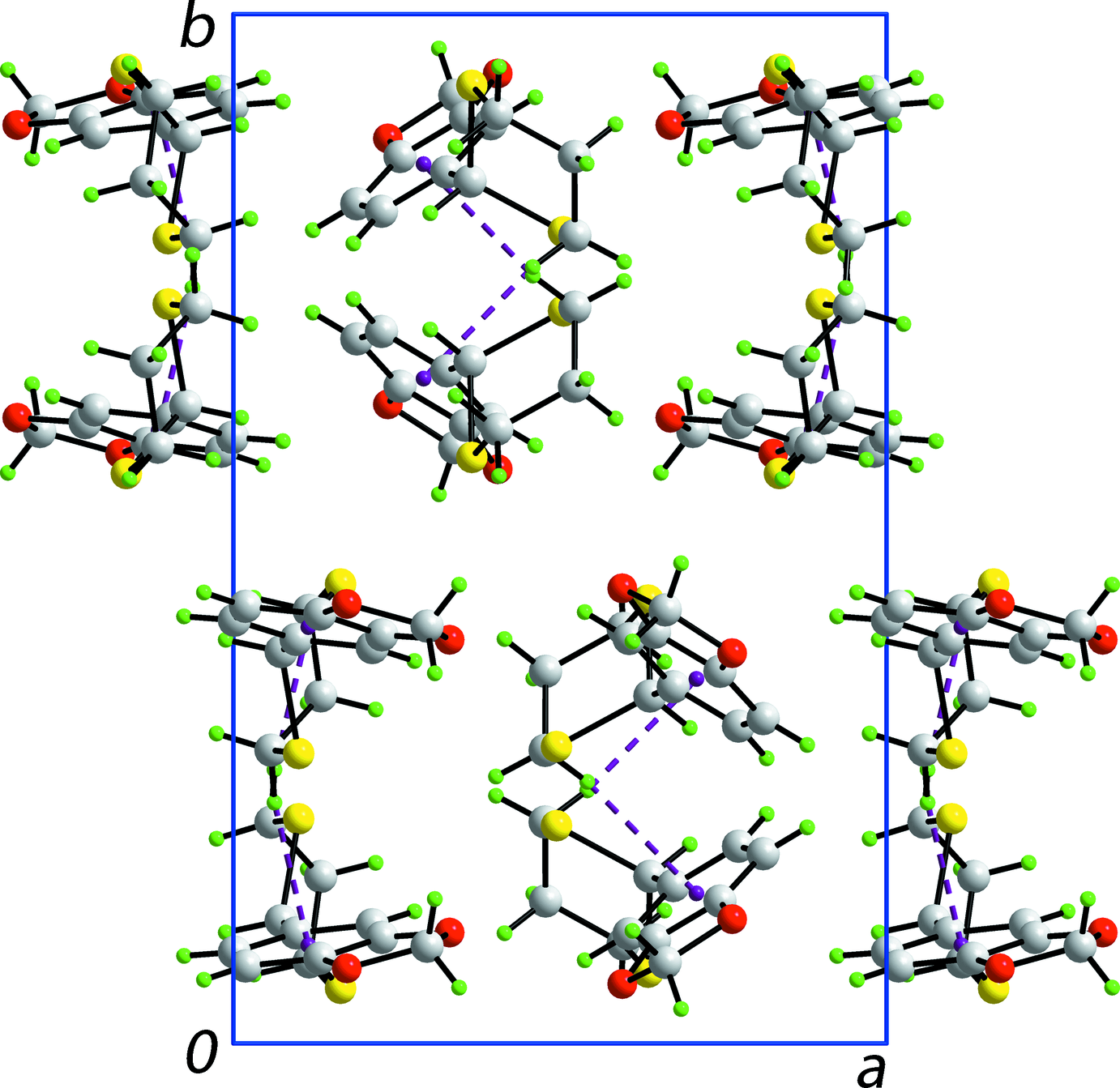

Supplement: Supplementary file 7 [file e-71-0o167-fig4.tif]
